# Who has anaphylaxis in Brazil? Validation of a questionnaire for population studies

**DOI:** 10.1186/s40413-017-0171-2

**Published:** 2017-11-08

**Authors:** Elaine Gagete, Lucilene Delazari dos Santos, Leticia Gomes de Pontes, Fábio Morato Castro

**Affiliations:** 1 Faculdade de MedicinaUniversidade de São Paulo (Medical School of University of Sao Paulo (USP), Rua Tenente Silvio Bestetti 590, Botucatu, SP Brazil; 2 CEVAP, Universidade Estadual Paulista – Botucatu (São Paulo State University - UNESP- Botucatu), Sao Paulo, Brazil

**Keywords:** Anaphylaxis, Epidemiology, Surveys and questionnaires

## Abstract

**Background:**

The incidence of anaphylaxis is increasing in several parts of the world; thus, determining the prevalence of the disease in a given region is important to understand the factors involved and to promote measures to avoid this type of allergic reaction. Aiming this objective, we validated an instrument for a population-basedstudy that assesses the prevalence of anaphylaxis in the Brazilian population.

**Methods:**

A questionnaire was generated in two variants - one for subjects seven years old or above (Group A) and another for children who were up to six years, 11 months and 29 days (Group B). The instrument was administered to patients with and without anaphylaxis. By allocating points, a score was calculated to differentiate subjects with and without the disease. After validation, the questionnaire was applied in the city of Botucatu (São Paulo state, Brazil), by randomly selecting houses and inviting residents to answer the questionnaire.

**Results:**

The questionnaire was reliable for identifying subjects with and without anaphylaxis in both groups, with a specificity and sensitivity above 90%. The prevalence of anaphylaxis in the pilot survey was 6.2% in Group A, however the evaluation was compromised in Group B by the low number of children below seven years of age due to random sampling of residences.

**Discussion:**

The prevalence of anaphylaxis in our pilot test (6.2%) was similar to major epidemiological surveys from several parts of the world, showing that anaphylaxis is not a rare disease. The instrument of the present work was suitable for this epidemiological survey and might be a good option for studying anaphylaxis in other populations.

**Conclusion:**

This instrument might be of particular value in places where researchers cannot access medical records to conduct similar epidemiological studies.

## Background

Anaphylaxis is a severe, acute and potentially fatal systemic allergic reaction triggered by hypersensitivity mechanisms. The diagnosis of anaphylaxis is made based on the NIAID/FAAN (The National Institute of Allergy and Infectious Disease and the Food Allergy and Anaphylaxis Network) 2005 consensus criteria [1]. A study of the accuracy of the NIAID/FAAN criteria for the diagnosis of anaphylaxis demonstrated that although their sensitivity is as high as 95%, their specificity is approximately 80% [2].

Because the incidence of anaphylaxis is reported to be increasing worldwide, studies on this disorder have become highly relevant. Three prior studies using data from the Rochester Epidemiology Project have studied the incidence of anaphylaxis among residents of Olmsted County, Minnesota, USA, over different time periods, which together span from 1983 to 2010 [3–5]. Their findings suggest that the incidence of anaphylaxis is increasing in that population, in both children and adults. Others have reported similar trends in the incidence of anaphylaxis overall and, more specifically, food-related anaphylaxis [6–8].

There are few epidemiological studies on anaphylaxis in Brazil [9, 10]. In this large country, the healthcare system is deficient in many aspects, and databases are not uniform and often not accessible.

Because of the difficult access to patient records in emergency or outpatient units, questionnaires administered directly to the population are important instruments, provided they are validated and used properly. With the aim to build an adequate instrument to study the prevalence of anaphylaxis in the Brazilian population, we created and validated this questionnaire to be used in epidemiological surveys.

## Methods

This novel instrument was generated according to recommendations by Portney and Watkins, 2009 [11] and Reichenheim and Moraes, 2008 [12], and the process followed the bellow described stages:

- Comprehensive bibliographic review of scientific material, published in Portuguese or English, to check whether other similar instruments had been developed and validated. Due to the lack of validated questionnaires with the desired characteristics, the next stage was pursued.
- Creating a questionnaire (Draft I) with questions in lay language, objective and grammatically correct sentences, using popular terms of everyday life. The questionnaire was conceived of as a mix between multiple-choice and short answer (open-ended) questions. Given the specific characteristics of the disease among small children compared to older children and adults, two variants of the questionnaire were generated: one for individuals who were seven years old or above (Group A) and another for children who were up to six years, 11 months and 29 days (Group B).
- Sending the questions of this Draft I to be analyzed by a body of ten judges, who were renowned professors in Allergology from several Medical Schools in Brazil. The following aspects were considered for each item: applicability, discriminative power, objectivity, biases, redundancy and classification capacity. The Likert scale was applied, with scores from 0 (“question entirely superfluous and inadequate”) to 5 (“question relevant and entirely adequate”). In addition to evaluating questions individually, the judges were also asked to grade the questionnaire as a whole from 0 to 5. Furthermore, each evaluator was encouraged to share their comments regarding the content and layout of the questionnaire. After the necessary corrections suggested by the judges, several trial tests were performed with small groups of patient until an updated version (Draft II) of the instrument was achieved.
- Draft II was then sent to ten other judges, who were also professors from Medical Schools in Brazil specialists in Allergy and Immunology. This second panel of judges evaluated each question in a more directed manner, based on the following criteria: 0: superfluous and inadequate question; 1: reasonable question; and 2: relevant and adequate question. This new group of judges was also asked to grade the questionnaire as a whole on a scale of 0 to 5 (where 5 indicates an excellent instrument). Their comments were considered and edited into a final version of the questionnaire.
- This final version was administered to patients (or their legal guardians) with and without anaphylaxis. Those with anaphylaxis had been previously diagnosed by specialists who used the Sampon et al. [3] criteria, known to be a gold standard for anaphylaxis diagnosis. The other group of patients had also been previously diagnosed with other allergic but non-anaphylactic diseases, such as asthma, urticaria, rhinosinusitis and other non-anaphylactic forms of food and drug allergies. All these patients were recruited from specialized outpatient services at the Medical School of University of Sao Paulo (USP), the Medical School of São Paulo State University (UNESP- Botucatu), or private allergy clinics in Botucatu. The allergological work-up to determine the etiological data for each patient had been previously conducted by these outpatient services, which included a thorough interview and in vivo and/or in vitro diagnostic allergological methods. In anaphylactic patients, severity of reactions was classified according to the Mueller [13] (Group A) or Brown [14] (Group B) scales. This study has later correlated questionnaire results that indicated anaphylaxis with the clinical results from outpatient services that had previously determined who in fact had this disease. The purpose of this method was to look for a positive correlation between the results from the questionnaire and the known clinical diagnosis.
- All patients were informed of the objectives of the study and signed the informed consent form previously approved by the Ethics Committees of both the above universities. The only identifiers in the questionnaire were date of birth, ethnicity and name initials. The researchers did know the full identity of interviewees, and this was necessary to double-check the questionnaire’s score with the patient’s diagnosis. To validate the questionnaire, researchers had to know whether a patient who had a high score was in fact someone who had had an anaphylactic crisis.
- Instrument validation: the analysis of the questionnaires began with the allocation of weights to the different questions to generate a score that divided the interviewees into groups with and without the disease based on a cut-off point that maximized sensitivity and specificity. The cut-off point was obtained by means of a receiver operating characteristic (ROC) curve. Weights were determined based on the relevance of symptoms for the diagnosis of anaphylaxis, and negative weights were assigned to exclude symptoms, evolution and treatments that are incompatible with the disease. All participants were asked to answer the same questionnaire approximately 60 days after their first response to assess the agreement rate of the answers.
- Pilot study: The questionnaire was administered in the population of Botucatu, which is a municipality in the Central West region of the state of São Paulo, with a population of 127,328 inhabitants, according to the Brazilian Institute of Geography and Statistics (Instituto Brasileiro de Geografia e Estatística –IBGE), census of 2010 [15]. Residences were selected according to a simple random sampling plan, and sample size was calculated based on 0.5% prevalence, 95% confidence and 0.05% sample error. Therefore, the sample size for Botucatu was at least 384 subjects. In each residence, individuals were invited to answer the questionnaire after signing the informed consent form. If they refused, a new residence was sampled until the total number of questionnaires was achieved. Participants (responding either for themselves or as legal guardians) were required to be above 16 years old in order to secure a high level of confidence in the answers provided. The interviewer was not to explain any question but could only read it to the interviewee, if he/she preferred to answer this way. If desired, the questionnaires were left in the home and collected a few days later. The final version of the questionnaires did not have any kind of personal identifier, so even in cases where the survey team came back to a house to pick up an envelope with a questionnaire, this information was not included when the results were compiled.

**Fig. 1:**
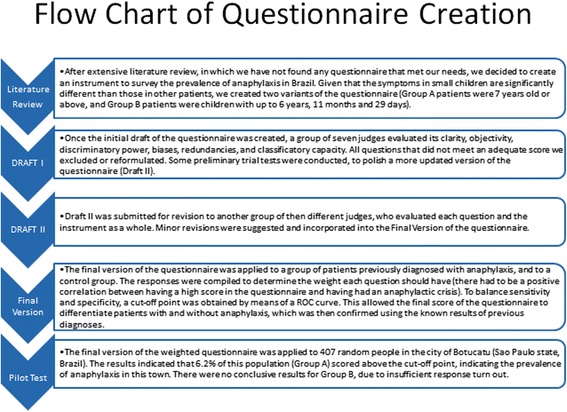
Summary of Methodology

A flow-chart with the summary of methodology is shown in Fig. 1.

## Results

Draft I of the questionnaire was composed of 22 questions with 111 sub-items. Of the ten judges who received this first questionnaire, eight returned it; however, one of them did not answer all questions and was therefore excluded. Nevertheless, his general comments were considered. Of the remaining seven judges, three graded the questionnaires as a whole with a score of 5, three of them with a score of 4, and one with a score of 3. None graded it with scores of 1 or 2.

In the analysis of individual questions by each of the seven judges, a modal score of 3 or above was considered acceptable. According to this, 92% of the questions had acceptable clarity (that is, 92% of the questions received a modal score of “3” or above from all seven judges). The other aspects were judged as follow: 72% received a grade of 3 or above for applicability, 80% for acceptable levels of biases, and 96% for acceptable levels of redundancy. Thus, there was little concern among authors regarding clarity, applicability, levels of biases and redundancy, given that all these criteria received good scores from the panel of judges.

The criteria that received scores bellow acceptable levels were discriminatory power, objectivity and classification capacity. Namely, only 37% of the questions received a modal score of 3 or above for their discriminatory power, 41% for objectivity, and 47% for classification capacity. It is important to underscore that judges evaluated all questions according to the six criteria mentioned above (applicability, discriminative power, objectivity, levels of biases, redundancy, and classification capacity). This means that any one question could have received a high score in one criteria and a low score in another. For example, among all questions in the questionnaire, only 41% received an acceptable score regarding its objectivity, so the authors had to decide how to address the problem of lack of objectivity in 59% of them. It was not possible to simply eliminate the questions that scored low in objectivity because many of them had received good scores regarding their applicability, clarity and/or acceptable levels of biases.

The authors then had to analyze individually each question, and decide whether to keep, modify, or eliminate it. Questions that had received a modal score of 3 or above in many criteria, and a low score in only a few, were either kept as they were or reworded. While questions that received scores below 3 in most criteria, were generally eliminated. This was a case by case process, discussed exhaustively amongst the authors. The reasoning behind this process was that it was important to create a questionnaire that overall had good levels of applicability, discriminative power, objectivity, classification capacity, and acceptable levels of biases and redundancy. Thus, if a question did not have a good score in one of these criteria, this would be compensated by other questions in the instrument having higher scores in the same criteria.

After several tests with the above corrections, the instrument (now Draft II) was reduced to 10 questions with approximately 80 sub-items for each variant (Groups A and B). As mentioned earlier, a second panel of ten professors judged the questionnaire again. Two sub-items from variant A and one from variant B received a score of 0 or 1 by at least two judges and were removed from the questionnaire. All of the other items and sub-items received scores of 2 by at least nine of the ten judges. The final score of the questionnaire as a whole was 4 (two judges) or 5 (eight judges). Thus, a satisfying final version was achieved.

A total of 199 patients were interviewed with this final version of these questionnaires and were classified as follows:

Group A (117 patients): 59 anaphylactic individuals (29 female - fem); 58 control individuals (35 fem).

Group B (82 patients): 26 anaphylactic children (11 fem); 56 control children (15 fem).

Tables 1 and 2 (A and B for the respective variants) shows a summary of the questions and their respective weights.

**Table 1:** Types of questions and respective weights in the questionnaire for patients in Group A

Type of question	Question	Weight
Medical diagnosis suggestive of anaphylaxis	2.0	10
Symptoms		
Rashes on the face and on the neck	3.1	1
Rashes on other parts of the body	3.2	1
Itchy scalp	3.3	2
Itching around the whole body	3.4	1
Swollen eyes	3.5	3
Swollen lips	3.6	3
Swollen tongue	3.7	3
Swollen genitals (private parts)	3.8	3
Other swollen body parts	3.9	3
Urticaria (hives)	3.10	3
Shortness of breath and/or cough and/or wheezing	3.11	1
Pallor	3.12	1
Cyanosis (purple extremities like nails, ear and lips)	3.13	4
Difficulties in speaking and/or changes in voice or crying	3.14	2
Difficulties in swallowing	3.15	4
Difficulties in thinking	3.16	2
Loss of consciousness or fainting	3.17	5
Cramps or stomach ache	3.18	1
Vomiting	3.19	3
Involuntary urination of fecal elimination	3.20	4
Other symptoms	3.21	1
Time from onset to full-blown crisis		
Less than 5 min	3.22–1	5
Between 5 and 30 min	3.22–2	4
Between 30 min and 2 h	3.22–3	3
Between 2 and 8 h	3.22–4	2
Between 8 and 24 h	3.22–5	1
More than 24 h	3.22–6	-8
Does not know/does not remember	3.22–7	0
Treatment compatible with anaphylaxis	4 and 5	2
Treatment incompatible with anaphylaxis	4 and 5	−4
How much time passed until back to normal		
Less than 12 h	6.1	1
Between 12 and 24 h	6.2	1
Between 24 and 72 h	6.3	1
Between 3 and 7 days	6.4	−2
More than 1 week	6.5	−5
Does not know/does not remember	6.6	0
Triggers		
Food	8.1	1
Medications	8.2	1
Insect (just *Hymenoptera*)	8.3	7
Latex	8.4	1
Exercise	8.5	1
Surgical or medical procedure	8.6	1
Dust	8.7	−3
Emotional	8.8	−5
Other	8.9	1
Does not know/does not remember	8.10	0

**Table 2:** Types of questions and respective weights in the questionnaire for patients in Group B

Type of question	Question	Weight
Medical diagnosis suggestive of anaphylaxis	2.0	10
Symptoms		
Rashes all over the body	3.1	2
Rashes on specific parts of body	3.2	2
Itching around the whole body	3.3	3
Swollen face	3.4	3
Swelling in other parts of the body	3.5	3
Urticaria (hives)	3.6	3
Shortness of breath and/or cough and/or wheezing	3.7	5
Voice or crying were husky or hoarse	3.8	5
Child was lethargic, did not react, was unresponsive	3.9	5
Diarrhea or softened stool	3.10	3
Vomiting	3.11	3
Time from onset to full-blown crisis		
Less than 5 min	3.12–1	5
Between 5 and 30 min	3.12–2	4
Between 30 min and 2 h	3.12–3	3
Between 2 and 8 h	3.12–4	2
Between 8 and 24 h	3.12–5	1
More than 24 h	3.12–6	−8
Does not know/does not remember	3.12–7	0
Treatment compatible with anaphylaxis	4 and 5	2
Treatment incompatible with anaphylaxis	4 and 5	−4
How much time passed until back to normal		
Less than 12 h	6.1	1
Between 12 and 24 h	6.2	1
Between 24 and 72 h	6.3	1
Between 3 and 7 days	6.4	−2
More than 1 week	6.5	−5
Does not know/does not remember	6.6	0
Triggers		
Food	8.1	1
Medications	8.2	1
Insect (just Hymenoptera)	8.3	7
Latex	8.4	1
Exercise	8.5	1
Surgical or medical procedure	8.6	1
Dust	8.7	−3
Emotional	8.8	−5
Other	8.9	1
Does not know/does not remember	8.10	0

(location in the text file: line 227)

It is worth noting that when the interviewee did not answer most of the questions, we have discarded the questionnaire. It is important that all items of every question be answered to ensure reliable result, especially for scored questions. When few items were skipped (less than five), we considered the answer to be “no” or “do not remember”, which are equally weighted as zero.

For complete questionnaire and raw dataset, please see online repository at https://figshare.com/s/5ae62c39e5dfeb518b2e

Note that:

I. Question 1 divides the population between those who had at least one previous episode of an allergic crisis in their life and those who did not. Thus, this question is qualitative and does not enter the score.
II. Starting with question 2, the interviewee or his/her legal guardian is asked to characterize the “worst allergic crisis he/she has ever had in his/her life”. The characterization of a single crisis (the worst) was necessary to avoid the summation of symptoms in the several eventually suffered crises, which we had noticed to be a trend among the interviewees during the trial testing phase of the instrument. The remaining questions (items related to triggers, outcome and treatment of the crisis) were formulated to remind the interviewee or the legal guardian to refer only to the worst suffered crisis.
III. Questions 7, 9 and 10 also did not enter the score because they are descriptive. Specifically, these questions refer to the number of severe allergic reactions suffered by the interviewee; whether the triggers were confirmed by tests or exams; and how much time passed between the trigger and the onset of the crisis, respectively.
IV. In question 8, each trigger is worth 1 point, in a non-additive manner, except for −2 points for house dust (mites) and −8 points for the emotional factor since we realized that these negative values were fundamental to distinguish between patients with asthma symptoms and those who overrated their symptoms, respectively. Aside from that, 7 points were added for patients who identified Hymenoptera insects as the trigger. This was necessary because some of the most severe cases of anaphylaxis, including blood pressure drop and collapse, were attributed to insects in a patient population that was otherwise oligosymptomatic.

Of the 117 patients from Group A who answered the questionnaire for the first time, 36 returned the second questionnaire by mail (30.8%). The mean agreement rate of the answers was 88%. For Group B (82 patients), 23 returned the second questionnaire (28%), and the mean agreement rate was 87%.

The results showed that mean scores were significantly higher in the anaphylaxis group compared to the control group (Table 3). The respective mean scores were:

**Table 3:** Comparison between patients with and without anaphylaxis

Age group	Controls (*n* = 58)	Anaphylaxis (*n* = 59)	p
Group A	9.9 ± 8.7 ^a^	36,6 ± 10,5 ^a^	< 0.001
	9.0(−12.0–27.0)^b^	35.0 (19.0–64.0)^b^	
–	Controls (*n* = 56)	Anaphylaxis (*n* = 26)	–
Group B	9.3 ± 7.9^a^	31.8 ± 7.3^a^	< 0.001
	9.0 (−8.0–27.0)^b^	31.0 (14.0–48.0)^b^	

^a^mean and standard deviation

^b^median and maximum- minimum range

•Group A: 36.6 +/− 10.5 and 9.9 +/− 8.7 and.

•Group B: 31.8 +/− 7.3 and 9.3 +/− 7.9 and.

For anaphylaxis severity, the mild anaphylaxis groups (Grade I and II in Group A and “mild” in Group B) exhibited statistically significant differences with respect to those of severe anaphylaxis (Tables 4 and 5). There was no significant differentiation between moderate and severe anaphylaxis in either group. The analysis for patients with Grade I anaphylaxis was compromised due to the negligible number of participants.

**Table 4:** Levels of anaphylaxis severity in patients from Group A

Grade I(*n* = 2)	Grade II (*n* = 12)	Grade III (*n* = 29)	Grade IV (*n* = 16)	P ^(1)^
28.28	24.7 ± 3.4	37.8 ± 7.8	44.5 ± 10.5	<0.001
–	24.5 (19–32)	36(29–57)	41.5(28–64)	–

(1) Kruskal-Wallis Test

Grade 2 < Grade 3, Grade 4 (*p* < 0.05; Dunn’s test for multiple comparisons)

**Table 5:** Levels of anaphylaxis severity in patients from Group B

Anaphylaxis severity level			
Mild (*n* = 10)	Moderate (n = 12)	Severe (*n* = 4)	P ^(1)^
26.8 ± 5.7	33.3 ± 5.2	40.2 ± 8.0	<0.001
28 (14–33)	32 (27–41)	41(31–48)	–

(1) Kruskal-Wallis Test

Mild <Severe (p < 0.05; Dunn’s test for multiple comparisons)

**Fig. 2:**
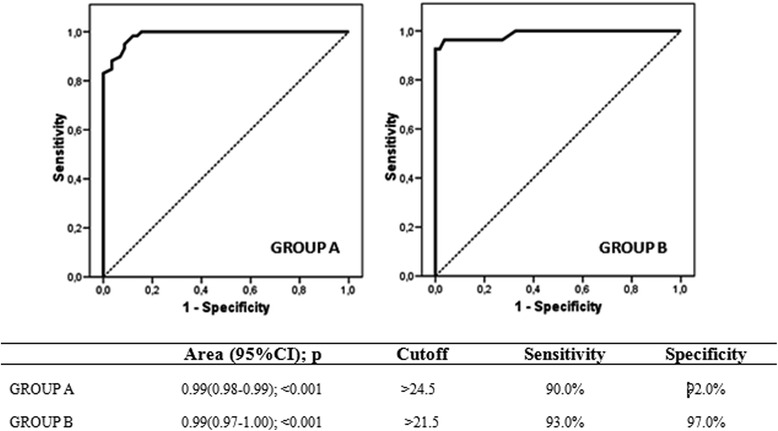
ROC curve for scores of the anaphylaxis diagnosis questionnaires (Group A and Group B)

The ROC curve analysis (Fig. 2) indicated that the cut-off points for the diagnosis of anaphylaxis were 24.5 for Group A (90.0% sensitivity, 92.0% specificity) and 23.5 for Group B (21.5% sensitivity, 97.0% specificity).

Considering that the NIAID/FAAN criteria [2] have a low specificity to diagnose anaphylaxis and that we sought to exclude from our study milder cases that could be misdiagnosed as other similar diseases, we randomly added 3 extra points to the cut-off value so that scores ≥28 and ≥25 were considered indicative of anaphylaxis in Groups A and B, respectively.

Cronbach’s alpha coefficient was 0.68 for Group A and 0.35 for Group B.

Once validated, the questionnaire was used in the city of Botucatu-SP, Brazil. A total of 407 questionnaires were completed, 387 by individuals from Group A (206 females) and 20 by individuals from Group B (8 females). Of the 387 respondents from Group A, 24 (6.2%) had a score of 28 or higher (suggestive of anaphylaxis). The reported causes for anaphylaxis were dipyrone (seven), shrimp or other crustaceans (five), insects (bees or wasps) (three), iodinated contrast (two) and unknown or unsure (seven). Group B, despite its small size, included four children with scores compatible with anaphylaxis (two to cow’s milk, one to bee venom and one unknown).

With respect to the behavior of the anaphylactic patients from the studied population, the majority exhibited more than one episode (16 interviewees, 66.6%), and nine patients (37.5%) reported four or more episodes. Sixteen patients (66.66%) did not seek specialized evaluation and were only seen in the emergency room.

## Discussion

Anaphylaxis is a medical emergency and among the most severe diseases dealt with by allergists. In a nearly global manner and for reasons not fully understood, the incidence of anaphylaxis has increased [3–5], as has its severity [6–8]. This explains the growing interest in epidemiological surveys to map the problem in several populations.

According to estimates, one out of 200 emergency care cases are caused by hypersensitivity reactions, ranging from mild urticaria to true anaphylactic reactions [16]. Epidemiological studies claim an estimated 50–2000 episodes of anaphylaxis per 100,000 persons, and, thus, approximately 2% of the population has already experienced at least one episode of anaphylaxis during their life [17].

The prevalence of anaphylaxis can be studied using data from different sources, such as emergency services, public and private medical facilities, hospital admissions, and consultations in allergist offices. Thus, the different methodologies must be considered when comparing rates in different populations. One example of this difficulty is a systematic review that was conducted to assess the epidemiology of anaphylaxis in Europe. Although more than 5000 papers were retrieved, only 49 met the methodological criteria compatible with a comparative study. The latter found an estimated incidence of 1.5 to 7.9 episodes per 100,000 person-years in Europe [18].

There are other challenges to be faced in epidemiological studies on this disease. The lack of a common term for diagnosis is among the most relevant. The term “anaphylaxis” is not listed in the ICD (International Classification of Diseases). Huang et al. [19] searched several classifications of diseases in the ICD-9 suggestive of allergic and anaphylactic reactions in pediatric patient records from emergency services and cross-referenced them with the symptoms of these patients, concluding that diagnoses such as “allergic reaction not elsewhere classified” (999.3), “adverse food reactions” (995.7) and “allergic urticaria” (708.0) were, in fact, anaphylaxis. In the ICD-10, this problem persists because there is only a code for “allergy, not elsewhere classified” (T78.4), “personal history of allergy” (Z88.0 to Z91.0) and “anaphylactic shock” (T78.0, T78.2, T80.5 and T88.6). The ICD is currently being revised, and its 11th edition is expected to be published soon. This edition will have a disease code specific for anaphylaxis, included in the allergy and hypersensitivity disorders of the immune system. This will hopefully make this disease more explicitly and properly documented in medical records, which will in turn allow for further epidemiological studies. [20].

Properly documenting anaphylaxis is not the only challenge. The diagnosis of anaphylaxis itself, particularly regarding borderline cases, can be difficult, especially for general practitioners, who represent most urgent care and emergency services. One possible method to evaluate the incidence of anaphylaxis is by studying self-injectable epinephrine prescriptions [21]. However, this is only possible in places where this drug is available to the general population, which is currently not the case in Brazil. Furthermore, in a study about epidemiology in Latin America [9], it was observed that corticosteroids were used in 80.5% of the patients, antihistamines in 70.2%, and epinephrine in only 37.3%. In other words, the prescription of adrenaline is not yet a safe indicator to assess the prevalence of anaphylaxis in these countries, given that this medication is under-prescribed. This also indicates that medical professionals are also not yet fully trained to properly respond and treat an anaphylactic crisis.

Brazil also has another characteristic that imposes further challenges to reliable research in databases which is the fact that there are only few specialists in the field of Allergology. There are only 1465 allergists to serve a population of more than 200 million inhabitants [22]. This challenge persists in educational realms, given that not all medical schools have professors to teach these subjects. The largest concentration of public hospitals that offer allergy specialists is in Southwest Brazil (where this research was carried out). However, many of them only have such services for pediatric patients. Private services are also scarce. Botucatu (also in this region), is an exception in the national context because it houses one medical school with allergy and immunology specialists, and one private clinic run by a specialist. This particular situation has allowed for the researchers to pull interviewees from different out-patient services and yet have high confidence levels that this selection was not biased towards certain groups of patients because all of them had been equally diagnosed by trained professionals.

In view of the difficulties in accessing uniform and reliable databases, one option is to survey the population directly and inquire about the symptoms, treatments and outcomes experienced during the episode. Currently, the diagnosis of anaphylaxis is based on a set of symptoms [1] that are subsequently validated [2]. Positive and negative predictive values of 68.6% and 98.4%, respectively, have been found. This means that if patients do not fit the diagnostic criteria, they are highly unlikely to have anaphylaxis. However, if they do meet the criteria, chances are still relatively high that they do not really have this disease. Thus, although adequate in emergencies, where the risk of misdiagnosis has far more severe consequences for the patients, these criteria do not substitute a more thorough evaluation by an allergist for a precise diagnosis. If this occurs during medical assistance, chances of retrieving this information via a population-based survey are even lower, particularly if the respondents are asked to recall their symptoms and diagnosis. Furthermore, other challenges with questionnaires include the availability and interest of interviewees to collaborate with this type of survey, difficulty understanding the questions, and the low level of education among some population groups. Another detail that may indicate reduced sensitivity of the instrument is that the first question asks the interviewees to choose their worst allergic crisis and to only talk about that because anaphylactic crises are generally striking episodes in the lives of those affected and are frequently dramatic. However, one can imagine that in milder cases of anaphylaxis, the interviewee chooses the allergic reaction that caused the most distress, such as a severe asthma attack.

Despite the above difficulties, researchers have relied on symptoms recall questionnaires in several medical areas, with favorable results. The most well-known study of this type in the field of Allergology is the International Study of Asthma and Allergies in Childhood (ISAAC) [23–24]. Questionnaires have also been used by other researchers to study the prevalence of anaphylaxis. In Australia, one interviewer questioned parents by phone with a non-standardized instrument, and the index of anaphylaxis was 0.59%. Two-thirds of these parents reported that their kids did not use emergency medicine during anaphylactic episodes in schools [25]. Alonso et al. [26] conceived and validated a questionnaire to assess the recurrence of anaphylaxis, first by administering the instrument to 52 individuals who were diagnosed as anaphylactic and then by submitting their responses to analysis by experienced allergists. The agreement between clinical diagnosis and that obtained by the questionnaire (kappa index) was 0.4 (moderate), with 73.1% correct.

The instrument introduced here followed all stages recommended for the generation and validation of an epidemiological survey [11,12]. After the first construct, its evaluation by two teams of highly qualified professors at different stages of the process ensured that several experts expressed their opinions and helped correct each question to make the instrument more precise and adequate in its intended objective to either confirm or rule out the presence of anaphylaxis. The gold standard of diagnosis for known anaphylactic patients, namely, the criteria established by Sampson et al. [1], was used for the allocation of weights. In this way, a score was reached with an optimal cut-off point given by the ROC curve that represents an important tool for applying the instrument to large populations. The internal consistency of the questionnaire, analyzed by Cronbach’s alpha, was good for Group A and poor for Group B. Despite the index being largely used to evaluate reliability, it depends on how the measure is used and cannot be used in an isolated manner to evaluate the questionnaire as a whole [27]. In this questionnaire (Group B), the difficulty parents or caregivers have when trying to identify symptoms and distinguish them within a context of anaphylactic reaction is a challenge. Many diseases are confused in this age range, particularly among laypeople, since they exhibit very similar signs and symptoms, including dyspnea, coughing, wheezing, and skin rash. Thus, it is safe to assume that the questionnaire would have low reliability if it were based only on signs and symptoms. Allocating weights (to emphasize what is most suggestive of anaphylaxis and de-emphasize what is least suggestive) and valuing medical diagnosis (which has the highest value in the final score) are important parts of this instrument to overcome the difficulties in differential diagnosis, particularly within this age range (Group B). Supporting this, both groups exhibited sensitivity and specificity of the instruments above 90%.

The prevalence of slightly above 6% observed in the pilot study is within the means found in international studies. A recent review by Tejedor-Alonso et al. [28] describes the major epidemiological surveys from several parts of the world and found indices ranging from 0.02% in Wales to 15% in the USA. The index of the present work of 6.2% clearly shows that anaphylaxis is not a rare disease in the population of Botucatu, and most likely in the Brazilian population as well. However, the pilot study revealed that random sampling might result in an insufficient sample of children younger than seven years old. This might be due to population characteristics, with decreasing birth rates over the past years, thus indicating population aging. Future studies focusing on this age range, conducted in places such as nurseries and pre-schools, will provide better insight into the prevalence of anaphylaxis in this group.

Regarding the causes of anaphylaxis, medications were the most commonly cited, followed by food and insects. These data are consistent with Bernd et al. [10], who provided the first survey on the causes of anaphylaxis in Brazil, and with Sole et al. [9], who studied Brazil and several other countries in Latin America. In the present pilot project, it is striking that more than 37% of the patients exhibited four or more episodes of anaphylaxis, and that more than 66% did not seek specialized help after the reactions.

To our knowledge, this is the first validated questionnaire to study epidemiology in a general population. Future surveys with this instrument in other regions will be necessary to acquire a broader understanding of the prevalence of anaphylaxis in Brazil. Not having auto-injectable epinephrine in Brazil is a serious problem, and one of the strategies to address it is to tackle the lack of awareness regarding the impacts of this disease at a national level. Thus, studying this problem is an important step for three main reasons: raising awareness of the importance to better treat anaphylactic patients, improving education and training about this disease in Brazilian medical schools, and calling the attention of decision-makers responsible for Public Health in this country to expedite legislation facilitating the access to auto-injectable epinephrine for anaphylactic patients.

## Conclusions

We conclude that with a validated questionnaire one can achieve a better understanding of the epidemiology of anaphylaxis. Our pilot study in a medium size city in Brazil showed that anaphylaxis in that population is as prevalent as in many other parts of the world (6,2%). This instrument can be used in other Brazilian studies aiming at providing more reliable anaphylaxis prevalence in the country. Moreover, it might also be useful for studies performed in similar developing countries that face the same difficulties in accessing health care databases.

## Abbreviations

IBGE: Instituto Brasileiro de Geografia e Estatística
ISAAC: International Study of Asthma and Allergies in Childhood
NIAID/FAAN: The National Institute of Allergy and Infectious Disease and the Food Allergy and Anaphylaxis Network
ROC: Receiver operating characteristic
UNESP: Medical School of São Paulo State University
USP: Medical School of University of Sao Paulo
